# The Behaviour of the EUV Corona Before Flares in Regions with High Free Magnetic Energy

**DOI:** 10.1007/s11207-026-02691-5

**Published:** 2026-06-17

**Authors:** Louise Harra, Kanya Kusano, Yingjie Zhu, Krzysztof Barczynski, Adriana De-Sassi, Muriel Z. Stiefel, Ioannis Kontogiannis, Matthew J. West, David H. Brooks

**Affiliations:** 1 Physikalisch-Meteorologisches Observatorium Davos/World Radiation Center (PMOD/WRC), Dorfstrasse 33, CH-7260 Davos Dorf, Switzerland; 2 IPA, ETH Zürich, Hönggerberg campus, HIT J22.4, Wolfgang-Pauli-Str. 27, 8093 Zürich, Switzerland; 3 Institute for Space-Earth Environmental Research, Nagoya University, Nagoya, Japan; 4 ESA / ESTEC, Keplerlaan 1, 2201 AZ Noordwijk, The Netherlands; 5 FHNW, University of Applied Sciences and Arts Northwestern Switzerland, Bahnhofstrasse 6, 5210 Windisch, Switzerland; 6 Computational Physics Inc., Springfield, VA 22151 USA; 7 Mullard Space Science Laboratory, University College London, Holmbury St. Mary, Dorking, Surrey RH5 6NT UK; 8 National Institutes of Natural Sciences, National Astronomical Observatory of Japan, 2-21-1 Osawa, Mitaka, Tokyo 181-8588 Japan

**Keywords:** Flares, Dynamics, Magnetic, Magnetic fields, Corona

## Abstract

Understanding and predicting the trigger of solar flares has been an area of focus for many years. In this work, we analyze EUV coronal data from SDO/AIA and Solar Orbiter EUI EUV High Resolution Imager (HRI_EUV_) to determine whether regions with high free magnetic energy in the photosphere show related dynamics in the corona. We studied two large solar flares from the same active region on 19 and 20 March 2024. We found that the two identified magnetic regions with high free energy also had frequent small spatial scale EUV intensity changes as highlighted through the standard deviation of the imaging data. The standard deviation enhancement in these regions peaked at least two minutes before the GOES X-ray emission and was close in time to the peak of the non-thermal emission, providing an additional method to identify the early flare trigger location and start.

**Supplementary Information:**

The online version contains supplementary material available at 10.1007/s11207-026-02691-5.

## Introduction

Solar flares are the most energetic phenomena in the solar system. A main research goal has been to understand the flare process, which has been developed over the past few decades, resulting in the ‘standard flare model’ known as CSHKP, (Carmichael 1964; Sturrock 1966; Hirayama 1974; Kopp and Pneuman 1976). This model is based on magnetic reconnection that is triggered by a rising magnetic flux rope. This 2-D model is now extended to 3-D by Janvier et al. (2013).

One of the more challenging areas of flare physics is understanding the flare trigger. Various mechanisms have been proposed that include MHD instabilities such as torus (Török and Kliem 2007) or kink instability (Török and Kliem 2005), and magnetic breakout (Antiochos, DeVore, and Klimchuk 1999) or tether cutting (Moore et al. 2001) models. These scenarios allow the magnetic flux rope to rise and trigger magnetic reconnection resulting in the release of electromagnetic energy.

Many observational signatures of the preflare phase have been determined over the years. The locations of small scale EUV brightenings in the preflare period was analyzed by Warren and Warshall (2001). These were found to occur before a flare but not in the location of the subsequent flare footpoints themselves. These sudden EUV impulses that could be precursors to a flare are explored by Krista (2025) using an automated method. They analyzed chromospheric emission and found that precursors were detected for 93% of the flares during a 6 hour window before the start of the flare. Dissauer et al. (2025) analyzed transient brightenings to determine if the brightenings were there all the time in an active region or did they form before a flare occurred. They found that the brightenings configure themselves in a large cluster close to the future flare ribbon site. They are focused around regions with a strong polarity inversion line or an increased magnetic energy density. Outside of flaring, small-scale transient brightenings still occur, but the clustering is smaller.

Spectroscopic signatures in the corona show enhancements of non-thermal velocity in X-ray and EUV emission lines before solar flares. These are particularly interesting because increases occur often before the hard X-ray emission starts to rise and the peaks occur before the soft X-ray intensity peaks, providing early quantitative information on the flare process (Harra, Matthews, and Culhane 2001; To et al. 2025). These increases, measured in the corona, can start tens of minutes before the flare begins. The spatial locations of these enhancements are not well understood yet, but results from Harra et al. (2013) show that in the case of eruptive flares, the enhancements in non-thermal velocity are found at the location of the activation of the magnetic flux rope. Early spectroscopic signatures have also been observed in the chromosphere (Woods et al. 2017), with shifts appearing around 40 minutes before a flare begins. Using deep neural networks, this work was extended by Panos and Kleint (2020), where they found that they could identify preflare spectra 35 minutes before the flare begins. Syntelis et al. (2016) found pre-eruptive EUV signatures indicative of a heating flux-rope, namely, an increase in blue shifted emission and non-thermal velocities, as well as in the mean Differential Emission Measure (DEM).

The new era of high spatial resolution coronal imaging from the Solar Orbiter EUV High Resolution Imager (HRI_EUV_) has led to a new understanding of solar flares. Chitta et al. (2025) illustrates this well with a thorough analysis of the HRI_EUV_ data of a large flare with high spatial resolution (210 km) and high temporal resolution (2 s). They found that in the preflare phase, rapid reconnection events frequently occurred with timescales of only seconds. In this case, they observed weak reconnection events that led to strong reconnection events. This occurred along the filament, with reconnection signatures appearing along a few threads initially and then 2 minutes later along the full length of the magnetic flux rope. This is consistent with a magnetic avalanche model.

In many of the observational studies to date, small-scale changes lead to the flare trigger through an instability. In this work, we investigate regions of high magnetic free energy in an active region that produces two large flares within 24 hours. We explore the coronal imaging data to determine if and how the corona responds to high magnetic energy and how that changes during the flare process.

## Observations

We analyze data from the High Resolution Imager (HRI) telescope of the Extreme Ultraviolet Imager (EUI; Rochus et al. 2020) on board the Solar Orbiter spacecraft (Müller et al. 2020). The HRI_EUV_ data were observed during a Major Flare Solar Orbiter Observing Plan (SOOP) from 19 March 2024 20:00:02 to 23:59:46. We analyzed HRI_EUV_ data from 23:00:00 to 23:59:46. The pixel size of the HRI_EUV_ (174 Å filter) data is 0.5^′′^ px^−1^, corresponding to a projected spatial scale of approximately 158 km px ^−1^ at a heliocentric distance of approximately 0.43 AU.

Jitter and solar rotation in level 2 HRI_EUV_ images were removed by co-aligning a quiescent region within the entire field of view using the cross-correlation technique described in Chitta et al. (2022). In brief, we divided the entire image sequence into short segments of 5 frames, with the successive segments sharing a common frame. Then we co-aligned the images within each segment to remove the jitter.

The Solar Dynamics Observatory (SDO) Atmospheric Imaging Assembly (AIA) data in the 171 Å passband (Pesnell, Thompson, and Chamberlin 2012; Lemen et al. 2012) were used for the analysis of dynamics in the corona. AIA has a temporal resolution of 12 seconds and a spatial resolution of 1.5^′′^. We differentially rotated the images to the first image in the time series and performed standard deviations over different time periods to quantify changes in the corona in different locations. Six channels from SDO/AIA: 94, 131, 171, 193, 211, and 335 Å were used for DEM analysis.

We also analyzed SDO/Helioseismic and Magnetic Imager (HMI; Pesnell, Thompson, and Chamberlin 2012) data that consist of line-of-sight (LOS) magnetograms selected from the full-disk data and the vector magnetic field at the solar surface from the Space-weather HMI Active Region Patch (SHARP) dataset (Bobra et al. 2014) to calculate the non-potential magnetic field using the Fourier transform. We used magnetograms obtained with a temporal resolution of 45 s or 720 s, depending on the analysis we perform, downloaded from the Joint Science Operation Center (JSOC, http://jsoc.stanford.edu/HMI/Magnetograms.html). In all cases, the magnetic data have a pixel resolution of 0.5^′′^.

For the flare on 19 March 2024, the *Hinode* (Kosugi et al. 2007) EUV Imaging Spectrometer (EIS; Culhane et al. 2007) was also observing. EIS provided spatially resolved spectra in the corona and was in ‘flare trigger mode’ during the active region observations. This means that it observed the active region with a wide slot (with reduced spectral information) and when the intensity during the flare increased, the instrument switched to a sit and- stare mode in which the slit was pointed to the flare region and spectra were obtained allowing determination of the non-thermal velocity. This switch in modes occurred early in the flare at 23:24:34 UT.

## Analysis: 19 March 2024 23:24

**Figure 1:**
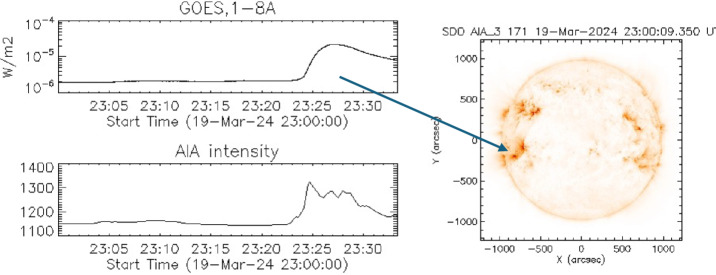
The top left plot shows the GOES X-ray 1 – 8 Å curve for the flare on 19 March 2024 peaking at 23:27 UT. The bottom left shows the average intensity light curve in AIA 171 Å of the flaring active region. The right hand image shows the full AIA image in the 171 Å filter for context. The blue arrow highlights the location of the flaring active region we study. A movie is available online ($March19\_20\_2024.mp4$).

On 19 March 2024 Solar Orbiter was located at a Heliocentric distance of $\approx 0.4$ AU at a Heliographic longitude and latitude of − 1.1^∘^ and − 7.6^∘^, respectively, approximately aligned with the Sun-Earth line. Active Region, NOAA13615, was located at S12E50 from the Earth’s perspective. The active region NOAA13615 produced an M2.1 class flare at 23:17 UT, shortly followed by an M7.4 class flare on 20 March 2024 at 07:23 UT. Figure 1 shows the location of the active region on the solar disk and the GOES X-ray intensity curve of the first flare.

**Figure 2:**
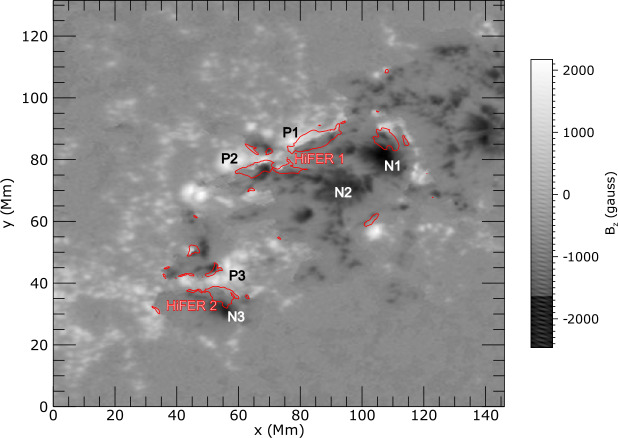
The HMI magnetogram is shown with white indicating positive flux and black showing negative flux (see colorbar). The main magnetic bipoles are marked as P1, N1, P2, N2 and P3, N3. The regions that have a non-potential magnetic field stronger than 1000 gauss are shown as contours, and these regions are defined as high free energy regions (HiFERs), labeled as HiFER1 and HiFER2.

The active region was analyzed using HMI on SDO in order to determine the free magnetic energy available in the region. This follows the method described in Kusano et al. (2020) based on Magnetohydrodynamic (MHD) instabilities (Ishiguro and Kusano 2017). This is driven by the Lorentz force acting on the current carrying magnetic loops, which is similar to the torus instability. Small-scale reconnection occurs between the current carrying magnetic loops over the polarity inversion line (PIL). Figure 2 shows the HMI magnetic data with the major positive and negative polarities highlighted. The regions that have high free magnetic energy are shown as contours, and these regions are labeled as ‘HiFER1’ and ‘HiFER2’.

**Figure 3:**
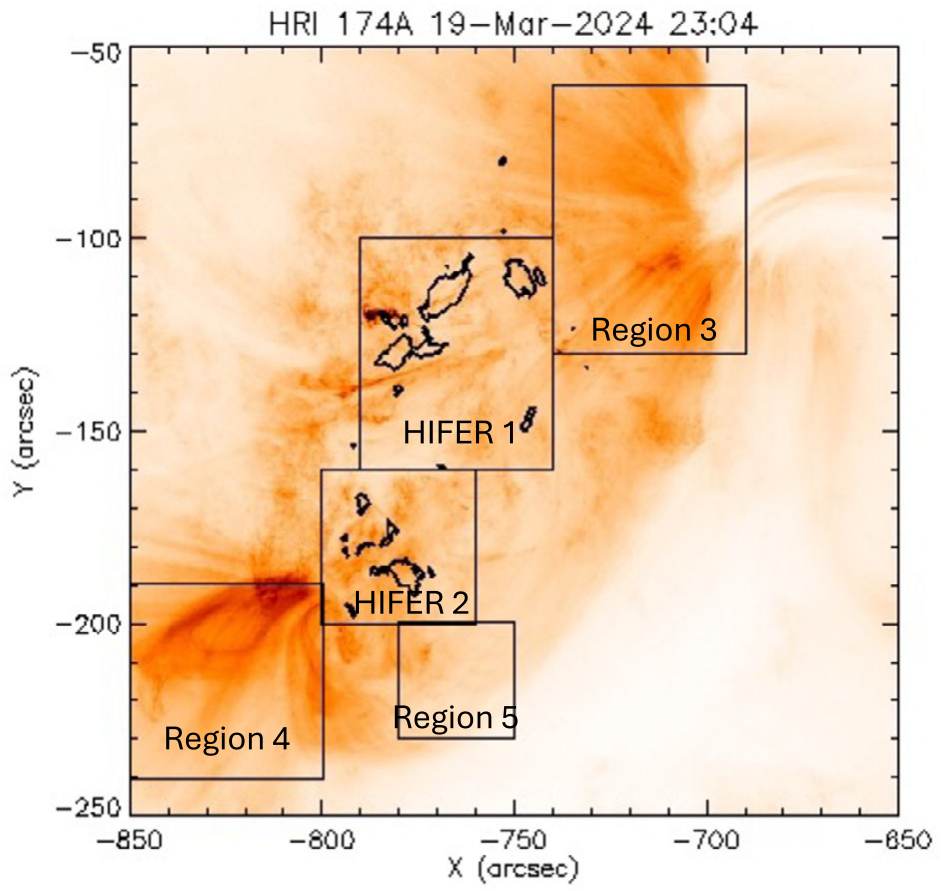
An EUV image from HRI_EUV_ before the flare on 19th March 2024 occurred. The black contours are illustrating the regions of high free energy (HiFER1 and HiFER2). The boxes shown highlight regions of interest that will be analyzed later. A movie of HRI data is shown online (hri_movie.mp4).

We analyze the EUV imaging data from both HRI_EUV_ and SDO/AIA to determine whether the regions of high magnetic free energy show any response at coronal temperatures. For this, we make use of the 171 Å filter on SDO/AIA and the 174 Å filter on HRI_EUV_. We selected two regions that are located at HiFER1 and HiFER2 to capture the corona above the regions of high magnetic free energy. In addition, we analyze other areas within the active region for comparison, and these are labeled region 3, 4, and 5, and shown on the HRI_EUV_ image in Figure 3.

**Figure 4:**
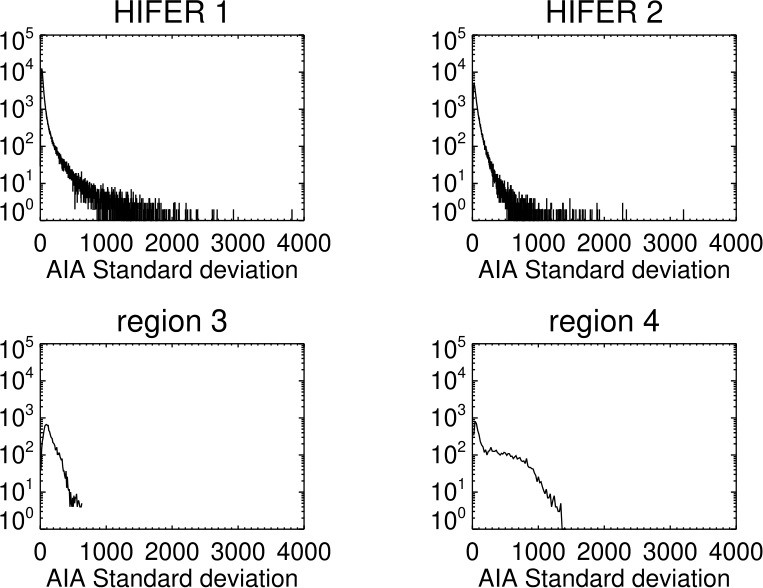
Histograms of the standard deviation of the AIA 171 Å intensity in the different regions, HiFER1, HiFER2 and regions 3 and 4. Both HiFER1 and HiFER2 show enhanced standard deviation during this time.

In order to assess the changes in the corona during the preflare phase of the first flare on 19 March, we determined the standard deviation of the intensity for each pixel in the AIA 171 Å image over the time-period from 22:47 UT to 23:20 UT, with the latter time chosen as it was before the GOES X-ray emission started to rise. For each region highlighted in Figure 3, we show the standard deviation histograms in Figure 4. Both regions HiFER1 and HiFER2 show standard deviations reaching more than 2000 DN/s, indicating strong variability, but they are not distinguishable from each other. Regions 3 and 4 have lower standard deviations, indicating lower variability. From this we can conclude that both regions with high magnetic energy also show enhanced dynamics in the corona. However, the HiFER2 region, which is the source of the first flare, is not distinguishable from HiFER1, the location of the second flare at the time period just before the first flare.

**Figure 5:**
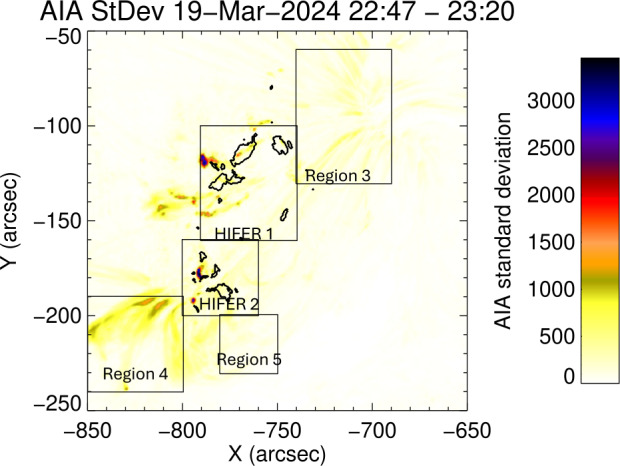
Standard deviation of the AIA 171 Å intensity indicating the variability of the emission in the active region. The different locations of interest are also shown.

The spatial distribution of the standard deviation is illustrated in Figure 5. Regions 3 and 5 do not have a significant enhancement of the standard deviation. HiFER1 (location of the second flare) and HiFER2 (location of the first flare) show enhancements at small spatial scales. Region 4 shows some enhancement along larger loops rather than in small locations.

**Figure 6:**
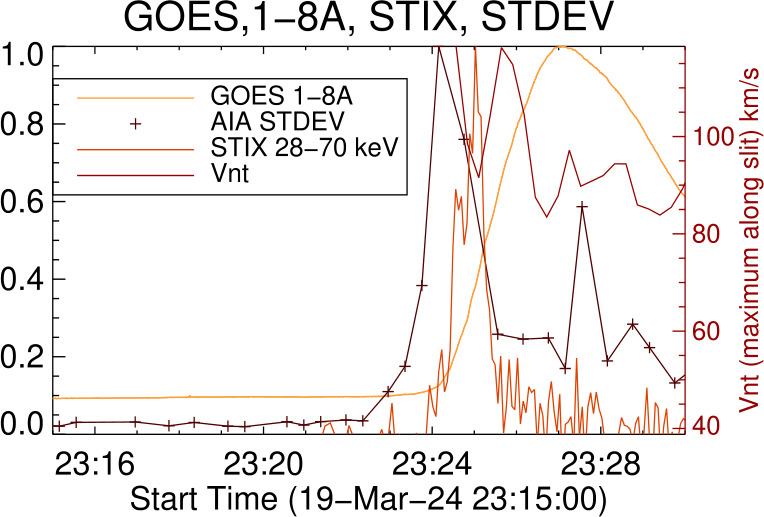
The figure shows the GOES 1 – 8 Å intensity normalised with time (orange line). The curve overlaid on this is the coronal standard deviation of HiFER2 with time (determined over 3 images with the time step taken as the middle time). Each time step is shown as a cross. The red curve shows the non-thermal emission from STIX. The Hinode EIS non-thermal velocity is shown as dark red and is at a peak at the first observing time at 23:24:34. The coronal standard deviation in HiFER2 peaks more than 2 minutes before the peak of the GOES X-rays and is close in time to the peak of the non-thermal emission from STIX.

The behaviour of the coronal dynamics via standard deviation is now determined with time to see if it increases before the flare begins. We looked at the standard deviation over 3 images in AIA (36 seconds) and tracked this over time. The behavior of the coronal standard deviation in HiFER2 shows a rapid increase, peak, and then decrease (see Figure 6). The time shown is the middle image time of the three images used to determine the standard deviation. The peak occurs at 23:24:09 UT, which is nearly 3 minutes before the peak of the GOES X-rays (at 23:27:05 UT). In fact, the standard deviation has nearly returned to the background level by the time the GOES X-ray curve peaked. The non-thermal hard X-ray emission from STIX (28 – 70 keV) peaks at 23:25:01 UT. Importantly, the coronal standard deviation of HiFER2 shows the earliest response, starting to rise measurably just after 23:23 UT.

**Figure 7:**
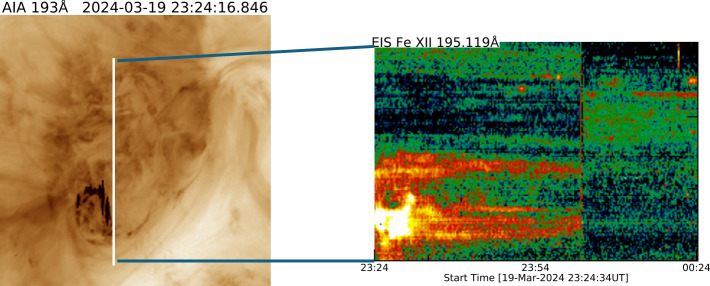
The left figure shows the AIA image at the time of the flare with the position of the Hinode EIS slit shown as a white vertical line. The right figure shows the Hinode EIS non-thermal velocity at each slit position with time (shown on the x-axes).

The Hinode EIS non-thermal velocity of the Fe xii 195.119 Å emission line is also shown. As stated before, EIS was triggered by the flare to observe in a slit mode that allows spectral resolution, and thus the determination of non-thermal velocity, from 23:24:34. By the time EIS triggered, the non-thermal velocity was already high with the first sit and stare measurement made. The maximum non-thermal velocity during this series was 118 km s^−1^ with a median of 37 km s^−1^. In Harra et al. (2013), they found a median of around 50 km s^−1^ and a maximum of 80 km s^−1^ for raster scans. The non-thermal velocity in this flare started to decrease after the end time of the figure shown at 23:32. The spatial location of the changes in the non-thermal velocity with time is shown in Figure 7. The non-thermal velocity showed the same behavior as has been seen many times before (To et al. 2025), where the peak in the non-thermal velocity occurred before the peak in intensity and was high during the peaks of the hard X-ray emission. However, the coronal standard deviation in HiFER1 does not change significantly during this time.

The Solar Orbiter and Hinode observations were also coordinated with the Interface Region Imaging Spectrograph (IRIS; De Pontieu et al. 2014). IRIS operated in large sit-and-stare mode (OBS 3443105703) from 19:38 UT on 19 March 2024 to 00:04 UT on 20 March 2024, recording 4984 images with a cadence of 3 s. The IRIS Slit-Jaw Imager (SJI), observing in the solar chromosphere (Mg II line), covered Region 3 and part of HIFER1 within its 118^′′^ × 119^′′^ field of view. A preliminary analysis of the standard deviation maps obtained from slit-jaw images for the same time range as AIA171 (22:47 – 23:20 UT on 2024-03-19) suggests that region HIFER1 contains several patches with significantly stronger standard deviation of intensity compared to Region 3. Since both regions were not observed we did not include IRIS analysis in this work.

In the next step, we used a differential emission measure (DEM) analysis to investigate the temperature distribution of plasma along the line of sight at three different times. The DEM was derived using observations from all six EUV channels of the SDO/AIA: 94, 131, 171, 193, 211, and 335 Å. For the inversion, we applied the regularized DEM method developed by Hannah and Kontar (2012). The analysis used SDO/AIA observations for three time steps between 23:05 and 23:24 UT on March 19, 2024 for the HiFER2 region. For each AIA image, we calculated the average intensity within the HiFER2 contours. The average intensities were then organized into groups, where each group contained intensities from the six AIA wavelength channels that observed the solar disk at the same time. Each group was treated independently and used as input to the DEM inversion code. This procedure was repeated for consecutive groups.

**Figure 8:**
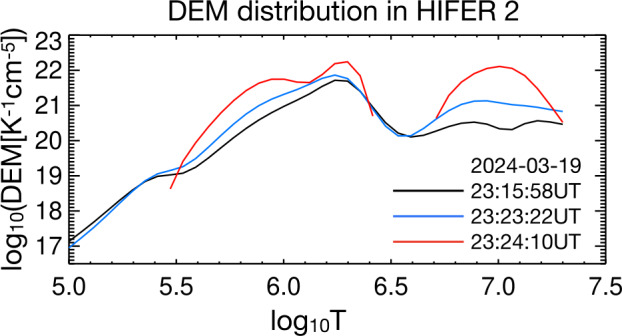
The figure shows the distribution of the Differential Emission Measure (DEM) for the HiFER2 region at three different time steps: a few minutes before the flare on the 19th March (black), when the AIA standard deviation starts to increase (blue), and immediately prior to the flare (red) as defined by the STIX non-thermal emission.

As a result, we obtained DEM distributions as a function of the logarithm of temperature, log DEM(log T/K), for three time steps for each HiFER2 region (Figure 8). Based on the analysis of the temporal evolution of the log(DEM) distribution with log(T), we find in the HiFER2 region a general temperature increase compared to the rest of the active region before the flare onset. The log(DEM) distribution shows two peaks: the first, corresponding to coronal emission, occurs at log T = 6.3, while the second, associated with the solar flare, peaks at log T = 7.0. Observations show that, several minutes before the flare, the coronal component dominates, with the flare component significantly weaker. However, just before the flare, when the AIA intensity and the standard deviation from SDO/AIA begin to increase, the flare-related component starts to rise as well and then starts to play a dominant role. In summary, the general enhancement in standard deviation in both HiFER1 and HiFER2 shows no enhancement in temperature - this occurs just before the flare occurs.

Next, we explore the EUV brightenings in the regions of interest. We have used an automated detection algorithm based on Berghmans et al. (2021) to identify small EUV brightenings in HRI_EUV_ (Auchère 2025). We used the first two scales of a dyadic à trous wavelet transformation of the images using a B_3_ spline scaling function. Pixels are accepted as brightenings if their wavelet coefficients exceed 45 times the noise level. The noise is estimated as $\sigma = \sqrt{\text{(read out noise)}^{2} + \text{(shot noise)}^{2}}$. Note that our threshold of 45$\sigma $ is much higher compared to the 5$\sigma $ threshold used in Berghmans et al. (2021). This adjustment was necessary because we are using the algorithm in an active region. We have only accepted brightenings that last for at least 2 frames and cover a minimum of two pixels in at least one frame. We omitted transforming the images into Carrington coordinates before applying the detection, since the region of interest is close to the limb, where remapping would introduce strong distortion. However, this means that the detected brightenings are always a projection onto the image plane.

**Figure 9:**
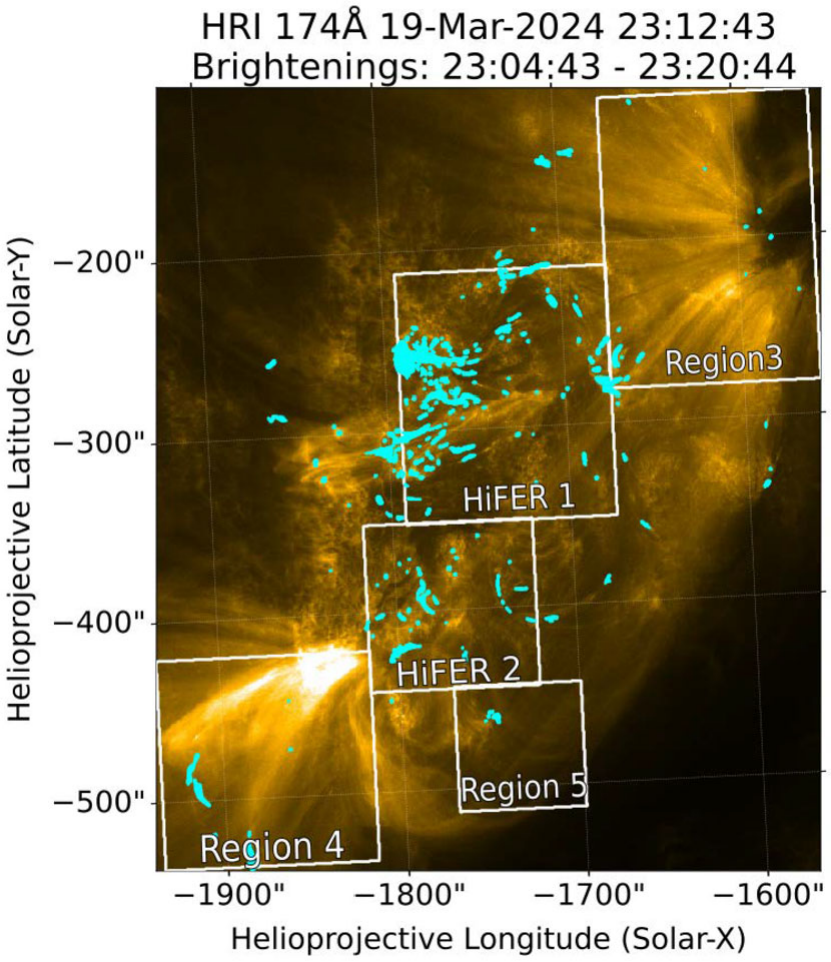
Detected EUV brightenings plotted with cyan contour on the HRI_EUV_ image at 23:12 UT. The brightenings were detected between 23:04:43 and 23:20:44 UT on 19 March 2024, expressed as Earth-received times.

All EUV brightenings detected in a 16-minute interval before the flare (23:04:43 to 23:20:44 UT) are plotted in Figure 9. The corresponding EUV brightening number densities in the image plane for the five regions are listed in Table 1. The brightenings have sizes between 300 km (2 pixels) and 4000 km. Both HiFER1 and HiFER2 show more brightenings than any other area in the active region. HiFER2, in which the first flare on 19 March was located, shows a density about 6 times higher compared to region 3 – 5. However, the brightening density in HiFER1, where the flare took place the next day on 20 March, is even higher (12 times).

**Table 1:** EUV Brightening density $[\mathrm{arcsec}^{-2}]$ detected in five different regions. The brightenings are detected in HRI_EUV_ between 23:04:43 UT and 23:20:44 UT on 19 March 2024.

HiFER1	HiFER2	Region 3	Region 4	Region 5
15⋅10^−3^	6.4⋅10^−3^	1.0⋅10^−3^	1.2⋅10^−3^	1.0⋅10^−3^

In summary, we find that the two regions in the active region that have magnetic free energy, also show enhanced dynamics in the corona through the standard deviation measurements. These enhancements in the standard deviation are seen in both HiFER regions. The change in the standard deviation with time shows a rapid increase in the early phase of the flare in HiFER2. This rises and peaks close in time to the non-thermal emission, but ahead of the GOES emission by more than 2 minutes. Both HiFER1 and HiFER 2 show higher EUV brightenings density than other areas of the active region. We now carry out the same analysis for the next flare that occurs in HiFER1.

## Analysis: 20 March 2024 07:23

**Figure 10:**
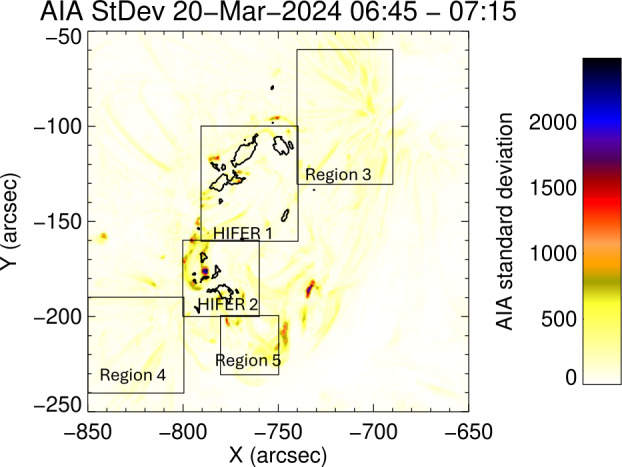
The standard deviation of the AIA 171 Å filter intensity on 20th March 2024 for 30 minutes before the flare starts. The 2 regions of high free magnetic energy are highlighted (HiFER1 and HiFER2) along with regions 3, 4 and 5 that are compared to the HiFER regions. The flare on the 20th March took place in region HiFER1.

We carried out the same analysis for the next M-class flare, which took place just over 7 hours later on 20 March 2024 at 07:23 UT. Figure 10 shows the spatial location of the standard deviation of the intensity per pixel in AIA 171 Å. As for the previous flare, the highest standard deviations were in regions corresponding to the highest magnetic free energy (HiFER1 and HiFER2). The other 3 regions had a lower standard deviation for a period of 30 minutes before the flare began. There is no significant difference between the standard deviation in HiFER1 and HiFER2. The flare on March 19th took place in the HiFER2 region, and the flare on March 20th took place in the HiFER1 region.

**Figure 11:**
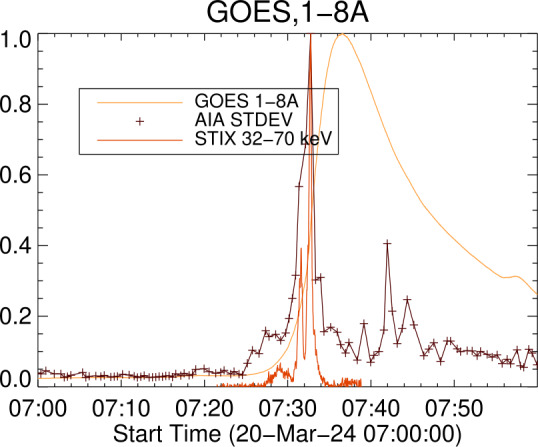
The normalised standard deviation of the AIA 171 Å intensity in HiFER1 (crosses) and the normalised GOES 1 – 8 Å X-ray intensity (orange) with time. The red curve shows the non-thermal emission from STIX.

The change in coronal standard deviation is shown in Figure 11. The peak of GOES 1 – 8 Å intensity occurred at 07:36:35 UT. The peak of the coronal standard deviation took place at 20-Mar-2024 07:32:45 UT (where the time used is the middle time of the 3 images that determine the standard deviation). The STIX non-thermal emission peaks at approximately the same time (07:32:45 UT). The results are similar to the flare on 19 March 2024. The coronal standard deviation peaks nearly 4 minutes before the GOES X-ray maximum occurs.

**Figure 12:**
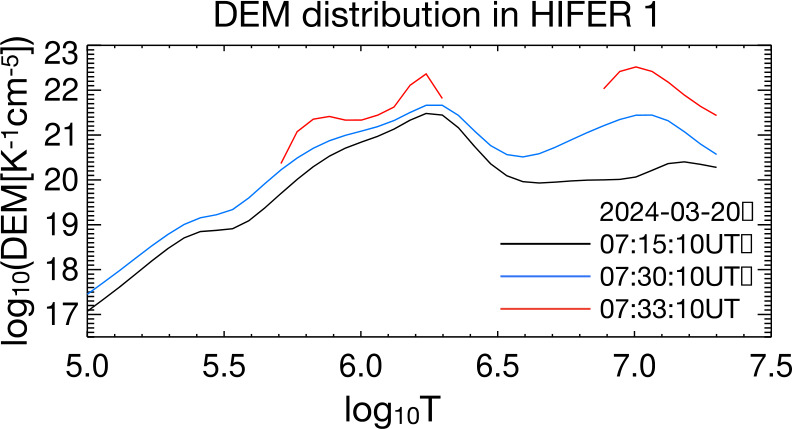
The plot illustrates the distribution of the Differential Emission Measure (DEM) for the HiFER1 region at three different time steps: a few minutes before the flare (black), when the standard deviation is increasing (blue), and immediately prior to the flare (red).

In the same way as in Section 3, we performed a DEM analysis focusing on the HiFER1 region observations between 07:15 and 07:33 UT (Figure 12). Based on the analysis of the temporal evolution of the log(DEM) distribution with log(T), we find common trends in both the HiFER1 and HiFER2 regions. Immediately before a flare, when the AIA intensity and the standard deviation from SDO/AIA begin to increase, the flare-related component also rises and gradually becomes dominant.

In summary, the coronal dynamics in the second flare behave in a similar way to the those of the flare on the 19th March. The coronal dynamics in the flaring region show enhanced standard deviation, more brightenings and higher temperature just before the flare begins.

## Analysis: What Are the Small-Scale Changes?

**Figure 13:**
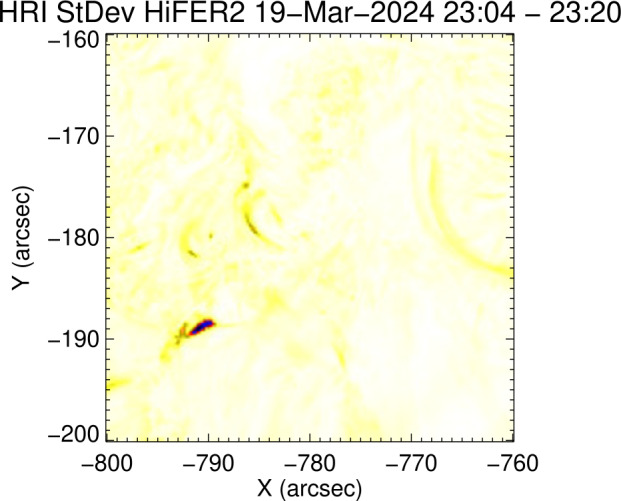
Standard deviation of HRI intensity, zoomed in on region HiFER2. There is a strong feature centred on -790^′′^, -188^′′^.

Before both M-classification flares, there was an increase in the standard deviation of the coronal intensities. We focus on one example of high coronal standard deviation in this region during the first flare on 19 March 2024, when we have HRI_EUV_ data available to observe the structure of these changes at high spatial resolution. Figure 13 shows the standard deviation using the high spatial resolution data from HRI_EUV_. The standard deviation was derived before the flare began and for a period of 16 minutes. This highlighted a small elongated region, which is around 5^′′^ long and less than 2^′′^ wide.

**Figure 14:**
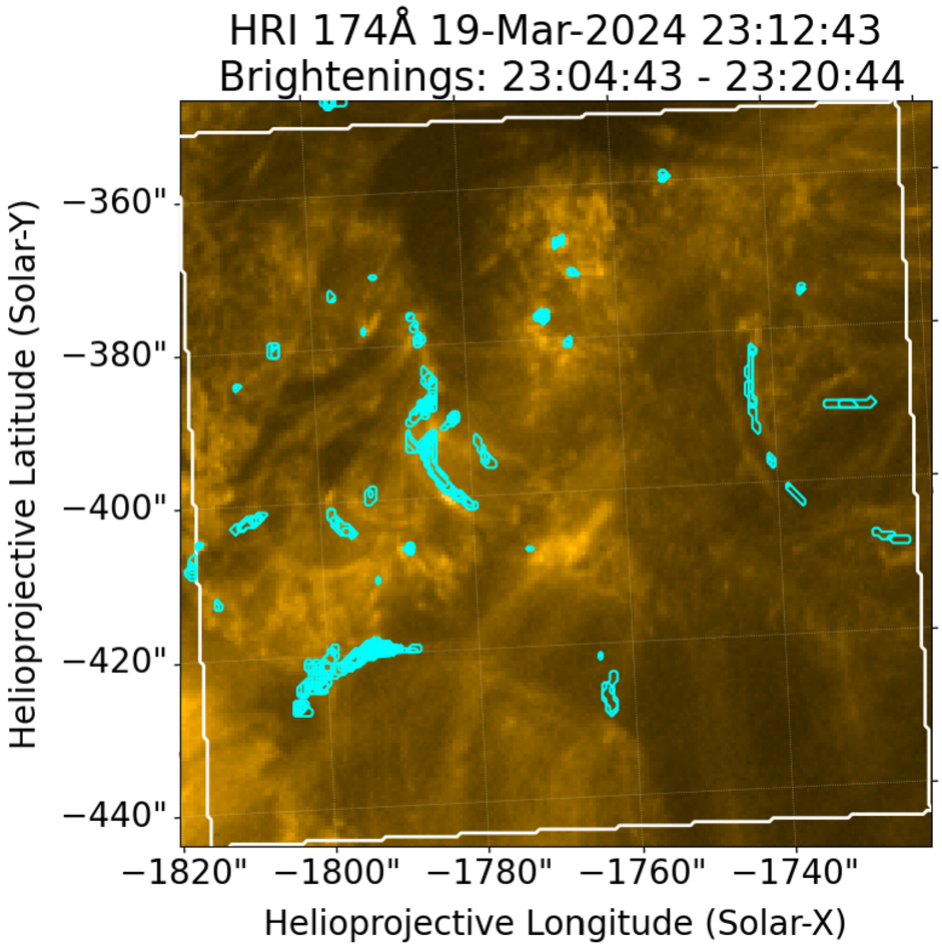
Zoom in on region HiFER 2 (white rectangle). The cyan contours show EUV brightenings plotted on EUV_HRI_. The brightenings were detected between 23:04:43 and 23:20:44 UT on 19 March 2024, expressed as Earth-received times.

**Figure 15:**
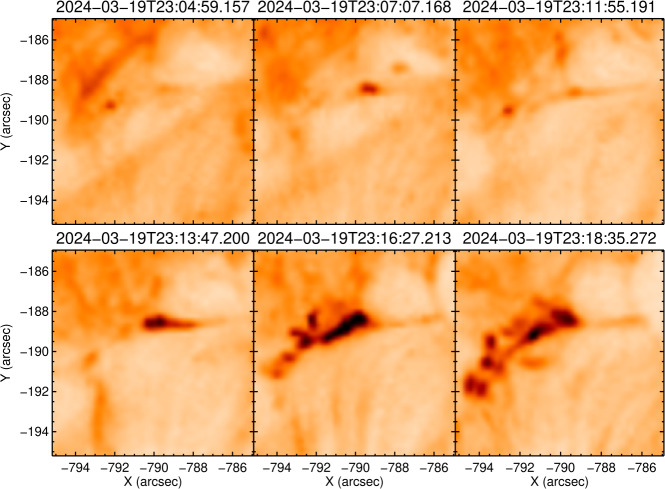
A time series of HRI data in the small region that shows enhanced standard deviation in HiFER2. The field of view is 10^′′^ × 10^′′^.

Figure 14 shows the same field of view with the EUV brightenings detected during the same time period. In the enhanced coronal standard deviation region, a series of small brightenings are detected (Figure 15). This figure is zoomed in to a 10^′′^ × 10^′′^ area that reveals small scale brightenings close to the limit of the spatial resolution of HRI_EUV_. Initially absent, small brightenings appear to form the observed structure, extending it to more than 5^′′^.

## Discussion

Understanding and predicting flares is a high priority goal, in particular, in the context of space weather predictions. Signatures that show enhancements of tens of minutes before the flare are seen in spectroscopic measurements in both the corona and the chromosphere. The current disadvantage of these spectroscopic measurements is that the spectral imagers have a small field of view and a slow cadence to cover just one active region and hence are not ideal for flare predictions.

Most flare predictions are currently based on the magnetic field as the source of the energy for flares (Kontogiannis 2023). The development of models has been carried out, e.g. Kusano et al. (2020) that can predict large flares based on MHD instabilities. In this paper, we explore whether regions determined by the models to contain high magnetic free energy show a response in the corona. We find that higher activity levels are seen in these regions of the corona. This provides another new diagnostic from the imaging data alone. The other important result is that these changes in dynamics, as measured through the standard deviation of coronal intensities, show an early rise and peak before the GOES X-ray emission. The peak occurs around 4 minutes before the peak in the X-rays, which is the standard method to define flares.

The next generation of EUV imagers will be on the ESA Vigil mission. This is the first ESA space weather mission to be launched in 2031 and will have an EUV imaging package (Joint EUV Coronal Diagnostic Investigation, JEDI) led by Don Hassler in SWRI. This will provide continuous monitoring of the Sun from the L5 Lagrange point, which will allow an early warning system for space weather impacts. JEDI has a space weather operational coronal imager in which this method could be used alongside the magnetic field measurements from the Photospheric Magnetic field Imager (PMI).

Our next steps are to do this analysis on a larger sample of active regions and determine whether combining the magnetic field data with the coronal imaging data can improve the prediction of solar flares.

## Supplementary Information

Below are the links to the electronic supplementary material.

(MP4 8.2 MB)

(MP4 33.1 MB)

## Data Availability

We make use of freely available data from NASA SDO and HMI, Hinode EIS and Solar Orbiter EUI. We state the data release used. The EUI data release 6.0 is available on DOI.
